# Insights on the Impact of Arbuscular Mycorrhizal Symbiosis on *Eucalyptus grandis* Tolerance to Drought Stress

**DOI:** 10.1128/spectrum.04381-22

**Published:** 2023-03-16

**Authors:** Sijia Wang, Ying Ren, Lina Han, Yuying Nie, Shuyuan Zhang, Xianan Xie, Wentao Hu, Hui Chen, Ming Tang

**Affiliations:** a State Key Laboratory of Conservation and Utilization of Subtropical Agro-Bioresources, Guangdong Laboratory for Lingnan Modern Agriculture, Guangdong Key Laboratory for Innovative Development and Utilization of Forest Plant Germplasm, College of Forestry and Landscape Architecture, South China Agricultural University, Guangzhou, China; State Key Laboratory of Mycology, Institute of Microbiology, Chinese Academy of Sciences

**Keywords:** arbuscular mycorrhizal fungi, *Eucalyptus grandis*, drought stress, osmotic regulation, antioxidation system, drought resistance genes, MAPK cascade genes

## Abstract

Drought stress has a negative impact on plant growth and production. Arbuscular mycorrhizal (AM) fungi, which establish symbioses with most terrestrial vascular plant species, play important roles in improving host plant mineral nutrient acquisition and resistance to drought. However, the physiological and molecular regulation mechanisms occurring in mycorrhizal Eucalyptus grandis coping with drought stress remain unclear. Here, we studied the physiological changes and mitogen-activated protein kinase (MAPK) cascade gene expression profiles of E. grandis associated with AM fungi under drought stress. The results showed that colonization by AM fungi significantly enhanced plant growth, with higher plant biomass, shoot height, root length, and relative water content (RWC) under drought conditions. Mycorrhizal plants had lower levels of accumulation of proline, malondialdehyde (MDA), H_2_O_2_, and O_2_^·−^ than seedlings not colonized with AM fungi. In addition, mycorrhizal *E. grandis* also had higher peroxidase (POD), superoxide dismutase (SOD), and catalase (CAT) activities under drought conditions, improving the antioxidant system response. Eighteen MAPK cascade genes were isolated from *E. grandis*, and the expression levels of the MAPK cascade genes were positively induced by symbiosis with AM fungi, which was correlated with changes in the proline, MDA, H_2_O_2_, and O_2_^·−^ contents and POD, SOD, and CAT activities. In summary, our results showed that AM symbiosis enhances *E. grandis* drought tolerance by regulating plant antioxidation abilities and MAPK cascade gene expression.

**IMPORTANCE** Arbuscular mycorrhizal (AM) fungi play an important role in improving plant growth and development under drought stress. The MAPK cascade may regulate many physiological and biochemical processes in plants in response to drought stress. Previous studies have shown that there is a complex regulatory network between the plant MAPK cascade and drought stress. However, the relationship between the *E. grandis* MAPK cascade and AM symbiosis in coping with drought remains to be investigated. Our results suggest that AM fungi could improve plant drought tolerance mainly by improving the antioxidant ability to protect plants from reactive oxygen species (ROS) and alleviate oxidative stress damage. The expression of the MAPK cascade genes was induced in mycorrhizal *E. grandis* seedlings under drought stress. This study revealed that MAPK cascade regulation is of special significance for improving the drought tolerance of *E. grandis*. This study provides a reference for improving mycorrhizal seedling cultivation under stress.

## INTRODUCTION

Plants are exposed to various external environmental stressors such as drought, heavy metal pollution, soil salinization, extreme temperatures, and other factors during the growth process (1, 2). Drought stress is one of the most severe abiotic stresses, limiting plant photosynthesis, affecting the water balance, and decreasing productivity, all of which seriously affect the growth and development of plants (3–6). Drought stress also leads to the accumulation of reactive oxygen species (ROS) (7), which could affect the normal physiological processes of plants, cause oxidative damage to cells, or even lead to programmed cell death (8). Fortunately, vascular plants have developed a wide range of adaptive mechanisms to cope with drought stress during the long process of evolution (9). Arbuscular mycorrhizal (AM) fungus symbiosis may be a good strategy to improve the drought tolerance of plants (10, 11).

AM fungi can form beneficial symbiotic associations with more than 72% of terrestrial plants (12, 13). Plants acquire mineral nutrients from AM fungi, and AM fungi obtain carbon compounds derived from the photosynthetic process to complete their life cycles (14–17). AM fungi not only improve the absorption of nutrients and water to promote plant growth but also help plants adapt to various environmental stresses, especially drought stress (18, 19). AM symbiosis provides a green, environmentally friendly, and sustainable way to improve the drought resistance of plants (20). A number of studies have indicated that AM fungi can enhance drought tolerance by changing plant physiological and molecular responses (11, 21). For instance, AM symbiosis can regulate plant osmotic adjustment abilities and antioxidant defenses, thereby reducing oxidative damage caused by ROS under drought stress (22, 23). Mycorrhizal plants show improved growth, photosynthesis, transpiration, water and mineral nutrient uptake capacities, aquaporin (AQP) activity, and water use efficiency in response to drought (5, 24–27). Additionally, AM fungi treatment up- or downregulated the expression of plasma-membrane-intrinsic proteins (PIPs) and the tonoplast-intrinsic proteins (TIPs) in response to drought (28, 29). Colonization by AM fungi can enhance symbiont water flow by regulating the expression levels of aquaporin genes in both host plants and AM fungi (30, 31). Although positive effects of AM symbiosis on plant resistance to drought have been reported, the underlying molecular mechanisms need to be explored deeply.

The mitogen-activated protein (MAP) kinase (MAPK) cascade plays a critical role in a series of cell external signal transduction and cell growth regulation processes in eukaryotes (32). A typical MAPK cascade consists of three protein kinases (MAP kinase kinase kinase [MAPKKK], MAP kinase kinase [MAPKK], and MAP kinase) (33, 34). Recently, with many plant genomes having been sequenced, the functions of the MAPK cascade genes during plant signal transduction and stress responses have been confirmed (35–37). It has been found that drought stress activates *AtMKK1*, which then induces the expression of *AtMPK4* and regulates stress-induced H_2_O_2_ by the catalase (CAT) pathway in response to drought in Arabidopsis thaliana (38, 39). The role of MKK4 in the response to drought was revealed by *mkk4* mutants, and the overexpression of MEKK18 significantly improved drought tolerance in *Arabidopsis* (40, 41). Recent studies showed that ZmMKK3, ZmMKK4, and ZmMPK1 from Zea mays can enhance drought tolerance by regulating abscisic acid (ABA) signal responses (42–44). Increased expression levels of MAPK cascade genes in mycorrhizal soybean roots were found to improve plant drought tolerance (45). MAP kinases activate OsWRKY30 to confer drought tolerance in rice (Oryza sativa L.) (46). The expression of MdMAPKs was induced under drought stress in *Malus domestica* (47). However, the MAPK cascade genes in mycorrhizal Eucalyptus grandis in response to drought stress require further study.

*Eucalyptus* tree species are some of the most widely planted hardwoods in the world. *Eucalyptus* has remarkable wood properties such as superior adaptability, outstanding diversity, and a high growth speed, all of which make it an important renewable resource in the world (48). Due to the influence of external environmental factors, *Eucalyptus* tree species are also suffering from drought stress. *Eucalyptus* species usually close stomata to reduce water loss through transpiration or regulate osmotic substances to balance the cell water content under drought stress (49–51). However, there are relatively few studies about how mycorrhizal E. grandis improves drought stress resistance. Therefore, we proposed the hypothesis that AM fungi may improve the tolerance of *E. grandis* to drought by regulating plant physiological and molecular responses and that the expression levels of MAPK cascade genes in *E. grandis* are influenced by AM symbiosis.

Here, we use a multidisciplinary approach that focused on antioxidant systems, osmotic adjustment, and the expression of *E. grandis* MAPK cascade genes. We characterized the *E. grandis* response to drought stress during the AM symbiosis process. In conclusion, our results showed that AM symbiosis positively affected drought tolerance in *E. grandis* by regulating physiological and molecular parameters.

## RESULTS

### Arbuscular mycorrhizal colonization promotes *E. grandis* tolerance to drought stress.

**FIG 1:**
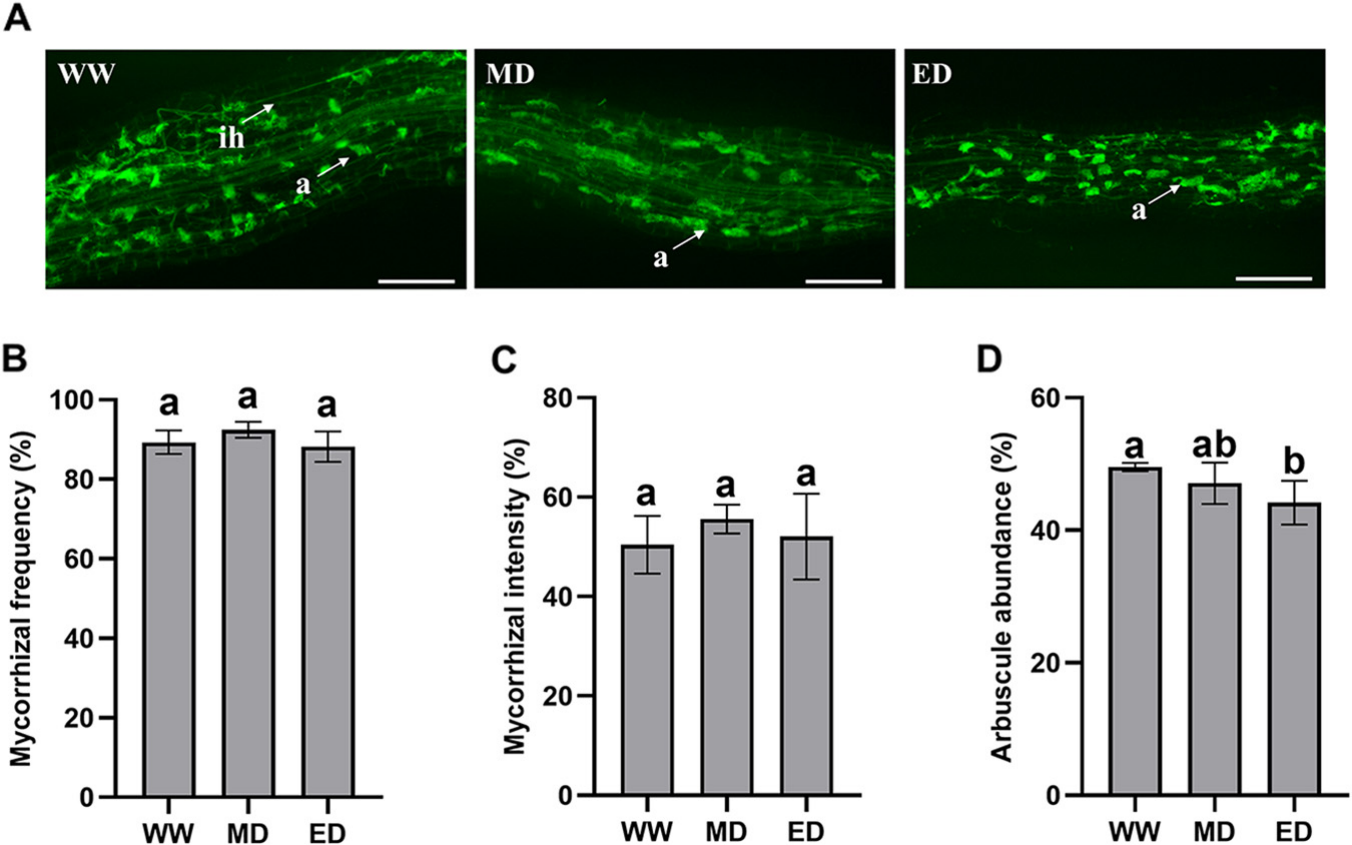
Arbuscular mycorrhizal colonization in *E. grandis* inoculated with *R. irregularis* under well water (WW), middle drought (MD), and extreme drought (ED) conditions. (A) Fluorescence microscopy images of *R. irregularis*-colonized roots of *E. grandis* after WGA488 staining under WW, MD, and ED conditions. a, arbuscule; in, internal hypha. Bars, 100 μm. (B to D) Total mycorrhizal frequency (B), mycorrhizal intensity (C), and arbuscule abundance (D) in *R. irregularis*-colonized roots estimated after WGA488 staining. The data are shown as the means ± SE from three biological replicates (*n* = 3). Different letters indicate significant differences at a *P* value of <0.05, according to one-way ANOVA and Tukey’s test.

**FIG 2:**
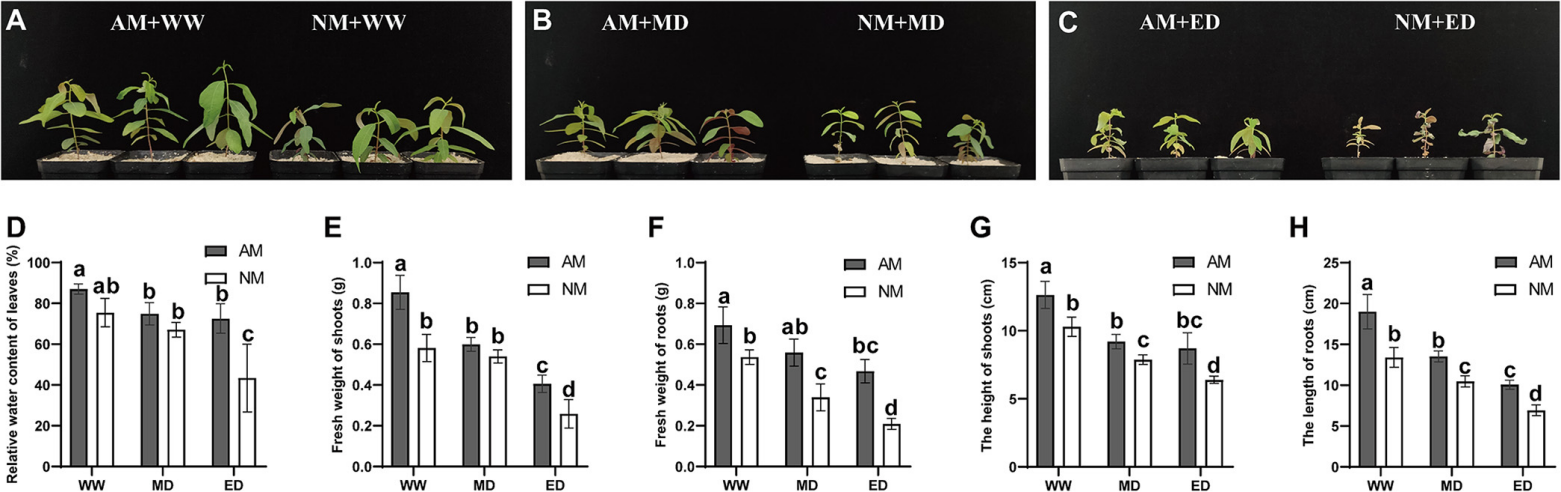
Effects of arbuscular mycorrhizal (AM) and nonmycorrhizal (NM) colonization on the growth, relative water content, fresh weight of shoots and roots, shoot height, and root length of *E. grandis* in response to well water (WW), middle drought (MD), and extreme drought (ED) conditions. (A to C) Growth performances of AM and NM *E. grandis* under WW, MD, and ED conditions. (D to H) Relative water content (RWC) (D), shoot fresh weight (E), root fresh weight (F), plant height (G), and root length (H) of *E. grandis* under different drought conditions. The data are shown as the means ± SE from three biological replicates (*n* = 3). Different letters indicate significant differences at a *P* value of <0.05, according to one-way ANOVA and Tukey’s test.

*E. grandis* seedlings colonized with Rhizophagus irregularis (AM) formed typical mycorrhizal structures under well water (WW), middle drought (MD), and extreme drought (ED) conditions. However, no mycorrhizal colonization was observed in nonmycorrhizal (NM) *E. grandis*. AM fungi and *E. grandis* established a symbiotic relationship, and numerous hyphae and typical arbuscular structures could be observed in the epidermal and cortical cells of the mycorrhizal *E. grandis* seedling roots (Fig. 1A). While there were no significant differences in the total percent colonization frequency (F%) and mycorrhization intensity (M%) during the different water treatments (Fig. 1B and C), the arbuscular abundance in *E. grandis* roots under WW conditions was higher than that under ED conditions (Fig. 1D). AM *E. grandis* grew better than the NM plants, and drought stress made *E. grandis* shorter, with etiolated leaves (Fig. 2A to C). The relative water content (RWC) is usually considered an important factor that reflects the water physiological conditions of plant tissues. Under WW and MD conditions, there were no significant differences in the RWC of plant leaves between the AM and NM plants, while the RWC of AM plants was significantly higher than that of the NM plants under ED stress (Fig. 2D). A comparison of AM and NM *E. grandis* revealed significant differences in *E. grandis* growth, including the plant fresh weight of shoots and roots, shoot height, and root length. The biomass, shoot height, and root length were significantly decreased in NM *E. grandis* seedlings compared with those in AM plants under MD and ED conditions (Fig. 2E to H).

### Effects of AM symbiosis on the contents of proline and other antioxidant substances (MDA, H_2_O_2_, and O_2_^·−^) in *E. grandis* in response to drought.

**FIG 3:**
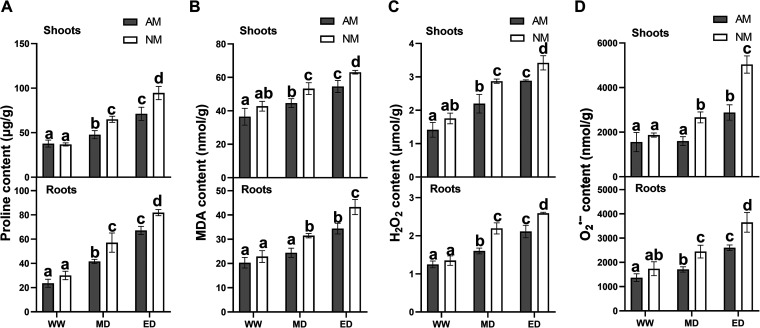
Proline (A), malondialdehyde (MDA) (B), hydrogen peroxide (H_2_O_2_) (C), and superoxide anion radical (O_2_^·−^) (D) contents of *E. grandis* seedling shoots and roots under different drought conditions. The data are shown as the means ± SE from three biological replicates (*n* = 3). Different letters indicate significant differences at a *P* value of <0.05, according to one-way ANOVA and Tukey’s test.

Reactive oxygen species (ROS) are inevitably induced when plants face environmental stress and have toxic effects on plants. To examine drought stress-induced ROS production in *E. grandis*, we assessed the proline, malondialdehyde (MDA), hydrogen peroxide (H_2_O_2_), and superoxide anion radical (O_2_^·−^) contents of *E. grandis* seedling shoots and roots. Under WW conditions, the proline, MDA, H_2_O_2_, and O_2_^·−^ contents in *E. grandis* seedling shoots and roots remained at low levels under both AM and NM treatments, and they were not significantly different. Under drought conditions, the proline content in shoots or roots increased significantly, while the AM plants showed lower proline contents than the NM plants (Fig. 3A). AM colonization significantly reduced the MDA contents in *E. grandis* shoots and roots compared with the NM plants in response to drought (Fig. 3B). The H_2_O_2_ and O_2_^·−^ accumulation patterns were similar to the proline and MDA accumulation patterns in *E. grandis*, with decreased H_2_O_2_ and O_2_^·−^ contents in mycorrhizal *E. grandis* compared to nonmycorrhizal *E. grandis* (Fig. 3C and D). These data suggest that AM fungal colonization regulates *E. grandis* antioxidant substances during drought stress.

### Response of antioxidant enzyme activity to drought stress in mycorrhizal and nonmycorrhizal *E. grandis*.

**FIG 4:**
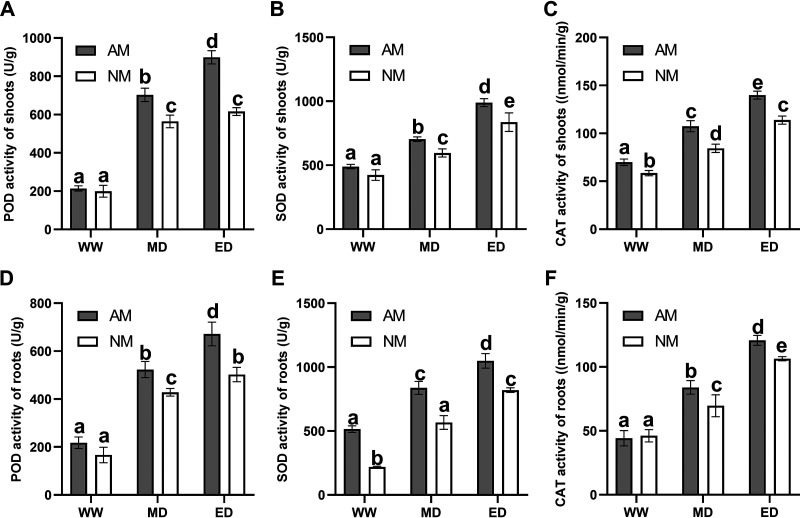
Effects of AM fungus colonization on the antioxidant activities of *E. grandis* under different drought conditions. Peroxidase (POD) (A), superoxide dismutase (SOD) (B), and catalase (CAT) (C) activities in shoots or roots of *E. grandis* seedlings are shown. Different letters indicate significant differences between treatments at a *P* value of <0.05, based on one-way ANOVA and Tukey’s tests. Error bars represent data from three biological replicates with SE values.

To investigate the effect of drought stress on the antioxidant system during AM symbiosis, we determined the contents of peroxidase (POD), superoxide dismutase (SOD), and catalase (CAT), which can reflect the activation levels of plant antioxidant enzymes. Under WW conditions, the POD activity in the shoots or roots of mycorrhizal *E. grandis* seedlings was increased, but it was not significantly different from that under NM conditions, while under MD and ED conditions, the POD activities in NM and AM seedlings were significantly different. The POD activity was much higher in AM plants than in NM *E. grandis* (Fig. 4A and D). Similar expression patterns were observed for the SOD and CAT activities in AM and NM *E. grandis* plants. Drought stress induced significant increases in the SOD and CAT activities in plant roots and shoots, and mycorrhizal *E. grandis* had higher activities than nonmycorrhizal *E. grandis* (Fig. 4B, C, E, and F). These results showed that AM fungus colonization had positive effects on POD, SOD, and CAT activities under drought stress.

### Relative expression levels of drought resistance genes in *E. grandis* and *R. irregularis*.

**FIG 5:**
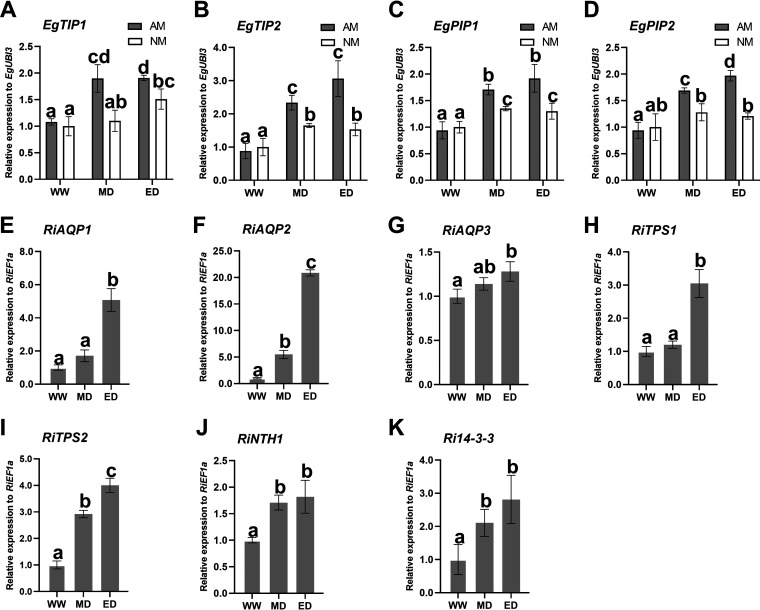
Relative expression levels of the stress response genes *EgTIP1*, *EgTIP2*, *EgPIP1*, and *EgPIP2* from *E. grandis* (A to D) and *RiAQP1*, -*2*, and -*3*; *RiTPS1* and -*2*; *RiNTH1*; and *Ri14-3-3* from *R. irregularis* in the mycorrhizal roots of *E. grandis* seedlings (D to K) under different drought stress conditions. Letters indicate significant differences between treatments at a *P* value of <0.05, based on one-way ANOVA and Tukey’s tests. Error bars represent data from three biological replicates with SE values.

To further investigate the molecular mechanisms of AM fungus symbiosis in response to drought, we analyzed the expression levels of drought resistance genes in *E. grandis* and R. irregularis using quantitative real-time PCR (qRT-PCR) technology. According to the released genome of *E. grandis* (48), plasma-membrane-intrinsic proteins (*EgPIP1* and *EgPIP2*) and tonoplast-intrinsic proteins (*EgTIP1* and *EgTIP2*) were identified using related PIP and TIP genes in other plants (28, 29). We also identified aquaporin (*RiAQP1*, -*2*, and -*3*), trehalose-6-phosphate synthase (TPS) (*RiTPS1* and -*2*), neutral trehalase (NTH) (*RiNTH1*), and *Ri14-3-3* genes in *R. irregularis* according to a previous study (52). There were no significant differences in the expression levels between AM and NM plants under WW conditions. However, under drought stress, the expression levels of *EgTIP1*, *EgTIP2*, *EgPIP1*, and *EgPIP2* were increased significantly, and the expression levels in the AM plants were highly upregulated compared with the NM plants (Fig. 5A to D). *RiAQP2*, *RiTPS2*, *RiNTH1*, and *Ri14-3-3* were expressed at high levels in response to MD and ED conditions compared to those under WW conditions, while increased expression levels of *RiAQP1* and *RiAQP3* were observed under ED conditions (Fig. 5E to K).

### MAPK proteins from *E. grandis* are conserved across plant species.

**FIG 6:**
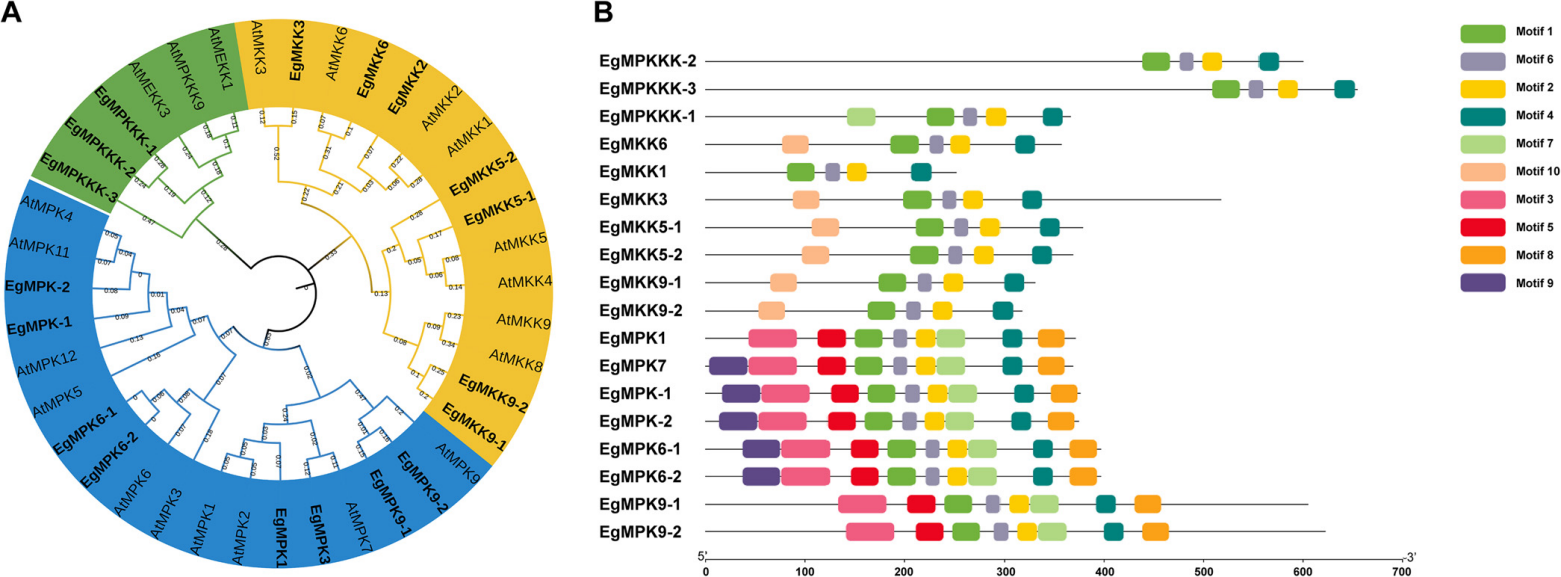
Evolutionary relationships and motif predictions of MAPK-related proteins in *E. grandis*. (A) Phylogenetic relationships between Arabidopsis thaliana and *E. grandis* MAPK-related proteins. The evolutionary history was inferred using MEGA11 software with the neighbor-joining method. The proteins in boldface type represent the MAPK cascade proteins in *E. grandis*. (B) Motifs of the MAPK proteins in *E. grandis*. The motifs were analyzed using MEME (https://meme-suite.org/meme/), and the figure was created using tbtools.

We initiated a search for the MAPK cascade genes in *E. grandis* using the related MAPK cascade genes in A. thaliana as reference sequences (53). The related MAPK cascade genes from *E. grandis* were identified by BLAST searches according to the released genome of *E. grandis* (48). To investigate the evolutionary relationships of MAPK cascade genes in *E. grandis*, we performed a phylogenetic analysis to construct a phylogenetic tree, which included 39 MAPK protein sequences from *E. grandis* and *A. thaliana* (Fig. 6). Studies on MAPK genes in *A. thaliana* have contributed significantly to research on MAPK cascades in other plants; therefore, we chose *A. thaliana* as a reference to analyze its homology with *E. grandis*. The *E. grandis* MAPK proteins were divided into three groups: MAPKKK, MAPKK, and MAPK. The EgMPKKK-1, EgMPKKK-2, and EgMPKKK-3 proteins are closely related to MAPKKK proteins of *A. thaliana*. Moreover, the EgMKK1, 3, 6, 5-1, 5-2, 9-1 and 9-2 proteins from *E. grandis* belong to the MAPKK group, and the EgMPK1, 6-1, 6-2, 7, 9-1, 9-2, -1, and -2 had high similarity to the MAPK group in *A. thaliana* (Fig. 6A). This result indicated the conserved evolutionary origin of the MAPK proteins in *E. grandis*. We also identified common motifs of *E. grandis* MAPK proteins using the Multiple Expectation Maximization for Motif Elicitation (MEME) website (http://meme-suite.org/tools/meme). In the group of MAPKKKs, EgMPKKK-2 and EgMPKKK-3 contained four motifs, while EgMPKKK-1 contained five motifs. Most members of the MAPKK group contained five motifs, except for EgMKK1, which contained four motifs. In the MAPK group, EgMPK1, EgMPK9-1, and EgMPK9-2 contained eight motifs, and the other members contained nine motifs (Fig. 6B). These results showed that the MAPK pathway of *E. grandis* may have the biological functions similar to those of the MAPK proteins of *A. thaliana*.

### Expression patterns of *E. grandis* MAPK cascade genes during AM symbiosis in response to drought stress.

**FIG 7:**
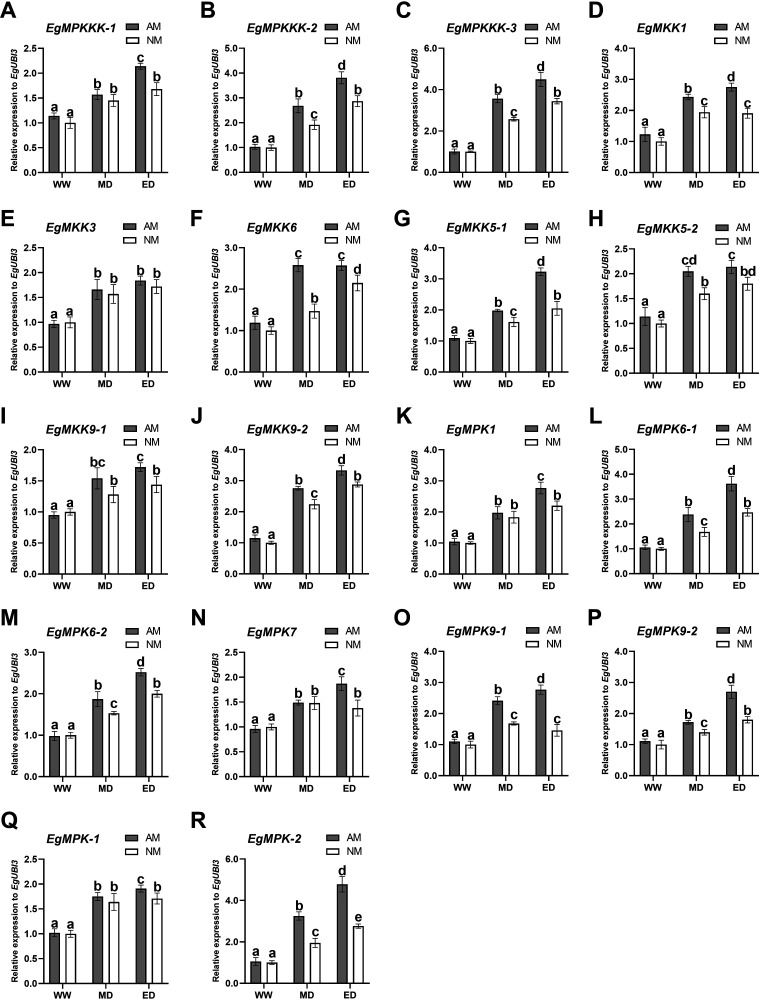
Expression patterns of MAPK genes in *E. grandis* under different water conditions. Shown are the expression patterns of MAPKKK (*EgMPKKK-1*, *-2*, and *-3*), MAPKK (*EgMKK1*, -*3*, -*6*, -*5-1*, -*5-2*, -*9-1*, and -*9-2*), MAPK (*EgMPK1*, *-6-1*, -*6-2*, -*7*, *-9-1*, *-9-2*, *-1*, and *-2*) genes of *E. grandis* in response to well water (WW), middle drought (MD), and extreme drought (ED) conditions. Different letters indicate statistically significant differences between treatments at a *P* value of <0.05, based on one-way ANOVA and Tukey’s tests. Error bars represent the results from three biological replicates with SE values.

In order to investigate the function of *E. grandis* MAPK cascade genes during AM symbiosis in response to drought, we analyzed the expression profiles of the MAPK cascade genes of *E. grandis* under WW, MD, and ED conditions. For all of the MAPK cascade genes, no significant changes were observed between AM and NM plants under WW treatment. However, the expression levels of these MAPK genes increased significantly under both MD and ED treatments (Fig. 7). Accordingly, the transcript levels of *EgMPKKK-1*, *EgMKK9-1*, *EgMPK1*, *EgMPK7*, and *EgMPK-1* were significantly upregulated in mycorrhizal roots under ED conditions (Fig. 7A, I, K, N, and Q), while under both MD and ED conditions, the expression levels of *EgMPKKK-2*, *EgMPKKK-3*, *EgMKK1*, *EgMKK6*, *EgMKK5-1*, *EgMKK5-2*, *EgMKK9-2*, *EgMPK6-1*, *EgMPK6-2*, *EgMPK9-1*, *EgMPK9-2*, and *EgMPK-2* were obviously upregulated in AM *E. grandis* compared with the control plants (NM) (Fig. 7). In conclusion, these results indicated that AM fungus symbiosis affected the expression of MAPK cascade genes in *E. grandis* in response to drought stress.

### Correlation of the regulation of *E. grandis* MAPK cascade genes and physiological parameters.

**FIG 8:**
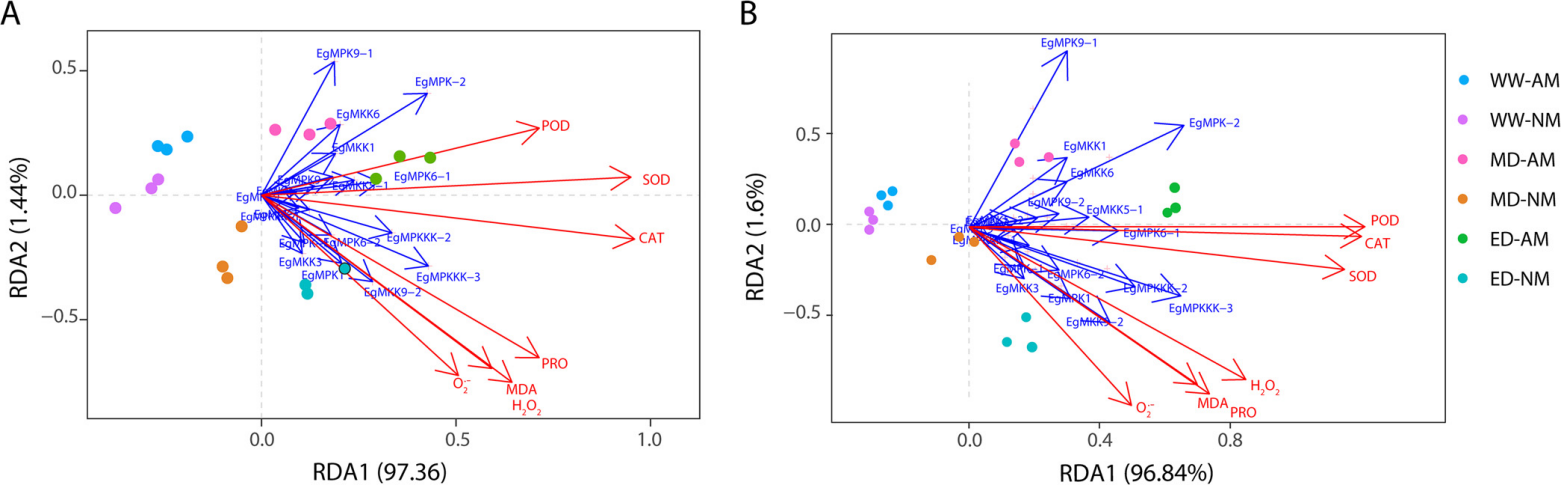
Redundancy analysis (RDA) of MAPK cascade gene expression and antioxidant indicators in *E. grandis* roots (A) and shoots (B) in response to different drought conditions. The colored dots represent different drought conditions. Dark blue, WW-AM; purple, WW-NM; pink, MD-AM; orange, MD-NM; green, ED-AM; light blue, ED-NM. The red arrows represent antioxidant systems, including POD, SOD, and CAT activities and MDA, H_2_O_2_, O_2_^·−^, and proline contents. The blue arrows show the expression levels of *E. grandis* MAPK cascade genes.

For the purpose of demonstrating the relationship between the expression levels of *E. grandis* MAPK cascade genes and the antioxidant system, we performed redundancy analysis (RDA) using RStudio. The results showed that the POD, SOD, CAT, proline, MDA, H_2_O_2_, and O_2_^·−^ physiological indicators in plant roots or shoots had acute angles with the first sorting axis, RDA1, and the arrows of them pointed toward the drought region, indicating that all of these indicators change in response to drought (Fig. 8). In addition, we found that the arrows of MAPK cascade genes were also distributed mainly in the drought region. *EgMPK9-1*, *EgMPK-2*, *EgMKK6*, *EgMPK6-1*, *EgMPKKK-2*, *EgMPKKK-3*, *EgMKK9-2*, and *EgMKK1* were the main genes that affected the antioxidant system of plant roots (Fig. 8A), while *EgMPK9-1*, *EgMPK-2*, *EgMKK1*, *EgMKK6*, *EgMKK5-1*, *EgMPK6-1*, *EgMPKKK-2*, *EgMPKKK-3*, *EgMKK9-2*, and *EgMPK1* mainly maintained the osmotic balance of plant leaves in response to drought (Fig. 8B), and *EgMPK9-1*, *EgMPK-2*, *EgMKK6*, and *EgMKK1* may be more likely to be expressed in mycorrhizal *E. grandis* under drought conditions in plant leaves and roots. *EgMPK9-1*, *EgMPK-2*, and *EgMPKKK-3* may be the main genes affecting the antioxidant system of *E. grandis* by improving drought resistance because they had the longest projection lengths on the first RDA1 axis in both leaves and roots (Fig. 8).

## DISCUSSION

Although AM fungi improve the drought resistance of plants, which has been confirmed in diverse plants species (5, 54, 55), very few of them have been researched in *E. grandis* trees; especially, the molecular features of MAPK cascade proteins in this tree species are not well known. In our research, *E. grandis* seedlings were colonized with *R. irregularis* to establish a symbiotic relationship. We demonstrate that AM symbiosis improves the drought tolerance of *E. grandis* by regulating the antioxidant system and upregulating the expression of some MAPK cascade genes.

### AM symbiosis improves physiological activity in *E. grandis* under drought stress.

In our research, the AM fungus *R. irregularis* and *E. grandis* seedlings establish a symbiotic relationship. We found that the mycorrhizal colonization level in *E. grandis* was not influenced by different water conditions (Fig. 1). This result is in agreement with the results of previous studies (31, 56, 57). We suspected that this was because drought treatment was performed at 42 days of colonization with AM fungi, which is not sufficient to influence the AM fungus colonization rate. Mycorrhizal *E. grandis* showed better performance, with increased fresh weight, plant height, and root length, under well water and drought conditions (Fig. 2). This might be the reason why AM fungi in the roots of *E. grandis* seedlings contribute to the formation of hyphal structures connecting with the soil. These hyphae can penetrate the soil and expand the root absorption area. Therefore, AM fungi can help plants acquire more water and nutrients to retain normal physiological functions in response to stressful conditions. Similar results were also described previously for other plant species (58, 59). Under ED conditions, the RWC of leaves in AM plants was increased compared with that in NM plants, which also showed that AM symbiosis helps plants absorb water.

Under stress conditions, ROS accumulate in large quantities in plants (60). Usually, plants initiate the antioxidant system to protect themselves from ROS accumulation when facing environmental stress conditions (61, 62). Antioxidant enzymes, including POD, SOD, and CAT, are considered important factors in reducing ROS accumulation (63, 64). In our experiments, the activities of POD, SOD, and CAT were significantly higher in *E. grandis* colonized with AM fungi than in nonmycorrhizal plants (Fig. 4). This trend was consistent with the patterns in the contents of active substances (MDA, H_2_O_2_, and O_2_^·−^) under drought stress. The contents of MDA, H_2_O_2_, and O_2_^·−^ were increased significantly in *E. grandis* seedlings, but their contents were lower in AM plants than in NM plants (Fig. 3). Water-stressed plants have been reported to accumulate proline to enhance the ability for resistance to drought (56, 65). We also verify that the proline content was higher in NM plants than in AM plants under drought stress conditions. A lower proline content suggests improved tolerance to drought (66, 67). Therefore, AM symbiosis maintains ROS accumulation in *E. grandis* seedlings at a relatively stable level by increasing antioxidant abilities under drought stress, thereby reducing damage to cells and alleviating serious damage caused by drought stress.

### Drought resistance genes of AM fungi and *E. grandis* during the regulation of mycorrhizal symbiosis in response to drought stress.

AM symbiosis can alleviate the negative effects of drought stress by regulating the expression of drought resistance genes such as the aquaporin-related genes. Previous studies have shown that AQP genes in Glomus intraradices transport water to host plants, and the expression levels of *GintAQPF1* and *GintAQPF2* were higher under water stress (30, 68). Trehalose-6-phosphate synthase (TPS) genes can improve resistance to drought in various plants and fungi (69). Neutral trehalase (NTH) was also an important factor during drought (70). The Ri14-3-3 protein in *R. irregularis* is essential for arbuscule formation (71). We also analyzed the expression levels of *RiAQP1*, *RiAQP2*, *RiTPS1*, *RiTPS2*, *RiNTH1*, and *Ri14-3-3* from *R. irregularis* in mycorrhizal *E. grandis*, which were identified previously by Wang et al. (52). We observed that the transcriptional levels of *RiAQP1*, *RiAQP2*, *RiTPS1*, *RiTPS2*, *RiNTH1*, and *Ri14-3-3* were increased significantly under ED conditions (Fig. 6E to K). Meanwhile, some studies also showed that AM symbiosis affected the expression of some PIP and TIP genes in plants (28, 29). Similar results were observed in our research, as increased expression levels of *EgPIP1*, *EgPIP2*, *EgTIP1*, and *EgTIP2* were found in mycorrhizal plants in response to MD and ED conditions (Fig. 6A to D). All of these results imply that AM fungus symbiosis may enhance plant drought tolerance by increasing the expression of drought resistance genes. The higher expression levels of all of these genes might lead to a better water uptake environment.

### The MAPK cascade regulates the physiological responses of mycorrhizal *E. grandis* to drought.

When plants are facing drought stress, they receive and transduce drought signals to regulate physiological and molecular responses to adapt to external stress. Fortunately, the MAPK signal transduction cascade is considered to have a critical role in the processes of regulating the immune responses to drought (53, 72, 73). Previous studies have reported that during AM fungus colonization of plant roots, the expression levels of MAPK cascade genes in AM fungi and plant roots increased significantly in response to drought (45, 47). In our study, 18 MAPK cascade genes were isolated from *E. grandis*, and they are conserved based on the phylogenetic relationships between *A. thaliana* and *E. grandis*, the motifs of which also showed evolutionary conservation in *E. grandis* (Fig. 5). The expression levels of related MAPK cascade genes in *E. grandis* were significantly upregulated in mycorrhizal plant roots under drought conditions (Fig. 7). AM symbiosis also has an impact on the expression levels of some MAPK cascade genes in other plant species such as Populus simonii × P. nigra (27), soybean (45), and Malus hupehensis (47). The MAPK cascade, as an essential regulator of antioxidant defense, can alter the expression profiles of antioxidant systems under diverse stress conditions (74). Previous studies reported that MAPK cascade genes directly regulate the antioxidant system by interactions with ROS (75). In addition, the expression of CAT1 is activated by MPK6 operating downstream of MKK1 during salt stress or after drought and ABA treatments (39, 76). Our results also showed that there is a high correlation between the expression of MAPK cascade genes and physiological parameters in *E. grandis* under drought stress. Antioxidant enzymes (POD, SOD, and CAT) may be related to the expression of MAPK cascade genes (*EgMPK6-1* and *EgMKK5-1*) in mycorrhizal *E. grandis* under drought conditions. Our research revealed that AM fungus symbiosis has a positive influence on the regulation of MAPK cascade genes in *E. grandis*. The MAPK cascade genes in mycorrhizal *E. grandis* seedlings may respond to drought stress by enhancing the antioxidant system and osmotic regulation. However, the potential functions and regulation of metabolism through these intricate interactions of MAPK cascade proteins remain unclear, for which further study is necessary.

In summary, we suggest that AM symbiosis alleviates the negative effects of drought stress on *E. grandis* seedlings by changing the plant’s physiology and MAPK cascade gene expression. Under drought stress, the expression levels of drought resistance genes from *E. grandis* and AM fungi were elevated significantly. The AM fungus *R. irregularis* improves the drought resistance of *E. grandis* by altering osmotic regulation, the antioxidant system, and the expression of MAPK cascade genes. Our study provides physiological and molecular evidence for the effects of AM fungus colonization on improving drought resistance and provides insights into the expression of MAPK cascade genes in *E. grandis*, which will provide new thoughts for improving mycorrhizal seedling cultivation under stress.

## MATERIALS AND METHODS

### Plant material, AM fungus colonization, and growth conditions.

*Eucalyptus grandis* seeds were provided by the Research Institute of Tropical Forestry (China Academy of Forestry, Guangzhou, China). *E. grandis* seeds were surface sterilized in 3% sodium hypochlorite for 20 min and then washed with sterile distilled water three times. The seeds were germinated in 1/4-strength Murashige-Skoog basal salt mixture agar medium at 25°C in the dark for 3 days, and the seedlings were then transferred to a growth chamber programmed for 16 h of light at 26°C and 8 h of darkness at 20°C for 14 days. After 14 days of cultivation, the seedlings were cultivated in small plastic pots (8 by 8 by 8 cm) with sterilized quartz sand. The roots of *E. grandis* seedlings were colonized with about 500 spores/plant.

The AM fungus used in this study is *R. irregularis* DAOM197198, which was purchased from Agronutrition, Toulouse, France.

### Drought treatments.

*E. grandis* plants grown in pot cultures were inoculated with (AM) or without (NM) *R. irregularis*. In the early stages of growth, all *E. grandis* seedlings were watered with a modified Long-Ashton (mLA) nutrient solution twice a week (77). After 42 days of inoculation, three kinds of water conditions were established in the experiments: WW (well water), with 75% field water capacity (FWC); MD (middle drought), with 50% FWC; and ED (extreme drought), with 25% FWC. The FWC was measured as described previously (78). There were 3 replicates per treatment. The soil moisture content was maintained by weighing at a fixed time every day. The drought treatment lasted for an additional 14 days; next, some mycorrhizal *E. grandis* root samples were used for colonization analysis, and the remaining root and shoot tissues were immediately frozen in liquid nitrogen and stored at −80°C for subsequent analyses.

### Quantification of mycorrhizal colonization.

Fresh roots of mycorrhizal *E. grandis* were immersed in 10% KOH at 90°C for 10 h, changing to a fresh KOH solution every 2 h. The roots were then neutralized in 2% HCl for 10 min and washed three times with sterile water. The samples were then stained with 5.0 μg/mL wheat germ agglutinin (WGA)-Alexa Fluor 488 conjugates (WGA488; Invitrogen, USA) for 30 min at 37°C. The roots were washed in 1× Hanks’ balanced salt solution without calcium, magnesium, and phenol red. The mycorrhizal levels of *E. grandis* were determined by WGA488 staining, and colonization was quantified using the MYCOCALC program as described previously (79). AM fungal structures were examined and captured using a confocal microscope (Y-TV55; Nikon).

### Plant relative water content, biomass, height, and root length.

When the *E. grandis* seedlings were harvested, the above-ground and underground weights, plant height, and root length were measured. Next, a part of fresh *E. grandis* leaves was weighed immediately to obtain the fresh weight (FW), the leaves were then soaked in deionized water for 24 h, the saturated weight (SW) was determined, and the leaves were dried for 48 h at 75°C to determine the dry weight (DW). The RWC in leaves was calculated as RWC = (FW − DW)/(SW − DW) × 100%.

### Analysis of active substances.

The proline content was measured at 520 nm with a UV-visible (UV-vis) spectrophotometer (Mapada, Shanghai, China) according to methods described previously by Bates et al. (80). Leaves and roots tissue (1 g) from fresh plant samples were weighed, frozen, and ground into a powder. One milliliter of an extract solution was added for ice bath homogenization, and the mixture was then centrifuged at 10,000 × *g* at 4°C for 20 min. The contents of the active substances MDA, H_2_O_2_, and O_2_^·−^ were then determined using an MDA kit, an H_2_O_2_ kit, and an O_2_^·−^ kit (Solarbio, Beijing, China), respectively, according to the manufacturer’s protocol.

### Analysis of antioxidant enzyme activity.

Fresh *E. grandis* leaves or root tissues (about 1 g) were ground with 1 mL chilled buffer containing 50 mM potassium phosphate buffer (pH 7.8), 1 mM EDTA, 0.3% Triton X-100, and 1% polyvinylpyrrolidone. The mixture was then centrifuged at 10,000 × *g* for 20 min at 4°C. Next, we conducted an analysis of the enzyme activity in the supernatant. Peroxidase (POD), superoxide dismutase (SOD), and catalase (CAT) activities were measured according to methods described previously by Beyer and Fridovich (81) and Amako et al. (82).

### Phylogenetic analysis.

According to the released genome of *E. grandis* (48), the MAPK cascade proteins of *E. grandis* were searched in the NCBI plant database using the BLAST program (83). The unrooted phylogenetic tree of *E. grandis* MAPK cascade proteins was generated using the neighbor-joining method in the MEGA11 program. The multiple-sequence alignment was performed using ClustalW, and the evolutionary distances were computed using the Poisson correction method. Bootstrap analysis was performed with 1,000 replicates. The motif distribution of MAPK cascade proteins in *E. grandis* was analyzed using an online website designed for this purpose (http://meme-suite.org/tools/meme), and a figure was created using tbtools (84).

### RNA isolation, cDNA synthesis, RT-PCR, and qRT-PCR.

Total RNA was extracted from *E. grandis* using the CTAB (cetyltrimethylammonium bromide) method (85), and first-strand cDNA synthesis was initiated using HiScript III reverse transcriptase (catalog number R323-01; Vazyme, Nanjing, China). Quantitative real-time PCRs were performed using ChamQ universal SYBR quantitative PCR (qPCR) master mix (Vazyme, Nanjing, China) in a 96-well real-time PCR system (Bio-Rad). All of the reactions were performed with three technical replicates of three biological replicates. The *EgUBI3* genes from *E. grandis* and the *RiEF1a* gene from *R. irregularis* were used as the internal controls for normalization. The relative expression levels of the genes were computed by the 2^−ΔΔ^*^CT^* method of relative quantification. A list of gene-specific primers used for qRT-PCR is given in Table S1 in the supplemental material.

### Statistical analyses.

All of the data were assayed using the SPSS Statistics 22 program. One-way analysis of variance (ANOVA) and Tukey’s test were used to compare the differences among treatments. The data are presented as the means ± standard errors (SE) for different replicates. Different letters in the figures indicate a significant difference at a *P* value of <0.05. Redundancy analysis (RDA) was performed by using RStudio.

### Data availability.

Sequence data from the manuscript can be searched in the NCBI Genome and GenBank libraries for the following MAPK cascade proteins: *E. grandis* EgMPKKK-1 (accession number XP_010063335.2), EgMPKKK-2 (accession number XP_039173142.1), EgMPKKK-3 (accession number XP_039172221.1), EgMKK1 (accession number XP_039166079.1), EgMKK3 (accession number XP_039157963.1), EgMKK6 (accession number XP_010049605.1), EgMKK5-1 (accession number XP_010063628.2), EgMKK5-2 (accession number XP_010034065.2), EgMKK9-1 (accession number XP_010069437.2), EgMKK9-2 (accession number XP_010036986.2), EgMPK1 (accession number XP_010060983.2), EgMPK6-1 (accession number XP_010066116.2), EgMPK6-2 (accession number XP_039172843.1), EgMPK7 (accession number XP_010033439.2), EgMPK9-1 (accession number XP_010032740.2), EgMPK9-2 (accession number XP_010036943.2), EgMPK-1 (accession number XP_010055090.1), EgMPK-2 (accession number XP_010055688.1), EgPIP1 (accession number XP_010043747.2), EgPIP2 (accession number XP_010043748.2), EgTIP1 (accession number XP_010037134.1), and EgTIP2 (accession number XP_010062508.1).
