# Supplementary material for: Insights on the Impact of Arbuscular Mycorrhizal Symbiosis on Eucalyptus grandis Tolerance to Drought Stress
Source: Microbiol Spectr. 2023 Mar 16;11(2):e04381-22. doi: 10.1128/spectrum.04381-22 (PMC10100883; doi:10.1128/spectrum.04381-22)
Supplement: Supplemental file 1 — Table S1. Download spectrum.04381-22-s0001.pdf, PDF file, 0.09 MB [file spectrum.04381-22-s0001.pdf]

Supplemental Table S1 PCR primers used for MAPK pathway genes

EgMPKKK-1-F	AGCAAATGAAGCGACTAGGG
EgMPKKK-1-R	ATGAGCAAGATGTCGAGAGC
EgMPKKK-2-F	GAGTTAGGGTTTTGAGTCGTAGG
EgMPKKK-2-R	TCGCAATTCTCACAATCTCCG
EgMPKKK-3-F	CAGCCTCCATATTCTCCCTTG
EgMPKKK-3-R	GAATTAAGTCTCGAGCGTCTCTAG
EgMKK1-F	ACCCCTTATCAAGCCAACAG
EgMKK1-R	CGATTCCCCTTTCCAATGACT
EgMKK3-F	TGACAAGCCAGACTCCAAATC
EgMKK3-R	ATCCTTGAACTCCATCAGTCG
EgMKK6-F	CGGGAGCACATATGACTATAGC
EgMKK6-R	CGCCTCCAAAAGCTCATAAAAG
EgMKK5-1-F	TGGTCTTTTCCTGTCGCTG
EgMKK5-1-R	ATCCCATGCCTTGTAACCG
EgMKK5-2-F	CATGACATCCAACGACATTGC
EgMKK5-2-R	ACCACTCATTCTGTAAGCCTC
EgMKK9-1-F	ACTACCTCCACTCCCTCAAG
EgMKK9-1-R	GATCTTGCTCACCCCGAAG
EgMKK9-2-F	TCATACACCGAGACATCAAGC
EgMKK9-2-R	CTTCGAGAGTCCTGCACATG
EgMPK1-F	CATTTTCGACCCATCAAAGCG

EgMPK1-R	TATTCGGATCATACAGCGGTG
EgMPK6-1-F	ATGTTAACGTTCGATCCCAGG
EgMPK6-1-R	TGTCGTGCAGTGAGTTTAGG
EgMPK6-2-F	AAGTTTCCCCATGTCCACC
EgMPK6-2-R	TGTCGTGCAGTGAGTTTAGG
EgMPK7-F	TCGTATCCCCTTGTCCTTTTG
EgMPK7-R	GGATCGGGAGAACTGAAAGTG
EgMPK9-1-F	AGATCGTCCAAGTGCTGAAG
EgMPK9-1-R	GACAGTTCCCTATCCACATTCG
EgMPK9-2-F	GAATCCAAACTCAGATGCGC
EgMPK9-2-R	TCGTCATTTACTTCAGCCTCC
EgMPK-1-F	GCTTTGTGCCACCCTTATTTG
EgMPK-1-R	GGATTGAATCTCAGAGTCTCCC
EgMPK-2-F	CGACTTGCTAGAGAAAATGCTTG
EgMPK-2-R	TTAATGTCGTGGAGGGATGC
EgPIP1-F	CACTGATCCAAAGCGAAACG
EgPIP1-R	TGAAGACTGCAAACCCTATGG
EgPIP2-F	GTTCCTCTACATCACGGTCTTG
EgPIP2-R	CGGCAGTGCAATAAACAAGG
EgTIP1-F	CTTCTCCGCGGGATCCTCTACT
EgTIP1-R	CCCAGATGACAGCGAGAAC
EgTIP2-F	TCTTTGTGTTTGCTGGTGTTG

EgTIP2-R	CGGAGATATTTGCACCCACTG
RiAQP1-F	GCATTCGGTGTAGGAGCTATC
RiAQP1-R	TGTCCACTAACTGCAATACCC
RiAQP2-F	GCTGTGCTTATTTTAGCTGGTG
RiAQP2-R	AACAATAGCGGGACCAAGAG
RiAQP3-F	CTCGGAGGTAGTGATGCAATAG
RiAQP3-R	ATGGCAGGATTCAATACTCCG
RiTPS1-F	TTTCTGGGCGTGATCTAACTG
RiTPS1-R	GAAACTTCCATGTTCTGCGC
RiTPS2-F	GATCGGCTGTTAGAGTACCATC
RiTPS2-R	GAATTAAGGCGTGTTAGTGATGC
RiNTH1-F	GATAGATGTGGAGGAAACTAAGCG
RiNTH1-R	ATGACCACCTGAATCAAGAGTACC
Ri14-3-3-F	GCTCAAACACGCGAAGATTAC
Ri14-3-3-R	AGGCAACAGACAAGAGATTACG
